# Lumican silencing ameliorates β-glycerophosphate-mediated vascular smooth muscle cell calcification by attenuating the inhibition of APOB on KIF2C activity

**DOI:** 10.1515/med-2023-0790

**Published:** 2023-09-07

**Authors:** Haibin Li, Chunyan Zhang, Qiang Liu

**Affiliations:** Department of Vascular Surgery, Ningbo Yinzhou People’s Hospital (The Affiliated People’s Hospital of Ningbo University), Ningbo, Zhejiang, 315040, China; Department Emergency, The First Hospital of Qiqihar’er City, Qiqihar’er, Heilongjiang, 161021, China; Department of Vascular Surgery, The First Hospital of Qiqihar’er City, No. 700, Bukui North Street, Longsha District, Qiqihar’er, Heilongjiang, 161021, China

**Keywords:** vascular calcification, lumican, apolipoprotein B, KIF2C

## Abstract

Adverse cardiovascular events are associated with vascular calcification (VC) process, where vascular smooth muscle cells (VSMCs) differentiate into osteoblastic phenotype and deposit hydroxyapatite crystals. Microtubule-associated protein kinesin family member 2C (KIF2C) expression is decreased obviously in VSMC during calcification induction. Accordingly, we investigate the role and potential mechanism of KIF2C on VSMC calcification. The effects of β-glycerophosphate (β-GP)/KIF2C/lumican (LUM) on calcification, calcium content, alkaline phosphatase (ALP) activity, calcification-related markers, Tubulin, the ratio of polymerized (Po) to free (Fr) tubulin, as well as levels of LUM, apolipoprotein B (APOB), and KIF2C were assessed by Alizarin red S staining, calcium assay kit, ALP assay kit, Western blot, immunofluorescence, and quantitative real-time PCR. The interplay between LUM and APOB was estimated using co-immunoprecipitation and immunofluorescence. As a result, β-GP promoted calcification of human VMSCs (HVMSCs) and repressed KIF2C expression. KIF2C overexpression reversed the effect of β-GP on HVSMCs. LUM silencing attenuated β-GP-induced promotion on HVSMC calcification and increased KIF2C expression by interacting with APOB. Collectively, LUM silencing can alleviate β-GP-induced VSMC calcification through mitigating the repression of APOB on KIF2C expression.

## 1 Introduction

Vascular calcification (VC) occurs in the intima (within atherosclerotic plaques) and/or media (usually associated with chronic kidney disease) [1], which elevates the risks of atherosclerotic plaque rupture and cardiovascular death [2]. Vascular smooth muscle cells (VSMCs) play an indispensable role in mediating VC through differentiating into osteoblast-like cells and producing matrix vesicles that result in calcium phosphate deposition in the vascular wall [3]. However, given the extremely complicated molecular mechanisms of VC, treatment of severe calcified vascular disease has only recourse to invasive transcatheter surgery [4]. So far, no drugs are available to effectively inhibit VC in clinical practice. Therefore, prevention as well as early diagnosis and treatment of VC to reduce cardiovascular adverse events and mortality has become an urgent clinical problem.

Dynamic remodeling of the microtubule cytoskeleton has been confirmed to play a crucial role in hyperphosphatemia-triggered VC. According to the study of Lee et al., inorganic phosphates can induce VC in mouse VSMCs, which is correlated with the change in tubulin dynamics and the induction of osteogenic signal [5]. To explore the mRNA changes in calcification-induced VSMCs, we analyzed the microarray data set (GSE74755) [6] from the Gene Expression Omnibus (GEO) database and found that the level of microtubule-associated protein kinesin family member 2C (KIF2C) was down-regulated obviously during calcification induction. The GeneCard database defined KIF2C as a microtubule-dependent molecular motor capable of depolymerizing microtubules at the positive end, and gene ontology annotation of KIF2C showed adenosine triphosphate hydrolytic activity and microtubule motility (https://www.genecards.org/cgi-bin/carddisp.pl?gene=KIF2C). The above information may reveal the important role of KIF2C in the disruption of microtubule stability during the calcification of VMSCs. Microtubule stabilization relieves VC by the inhibition of osteogenic signaling and matrix vesicle release [5]. Besides, the existing reports confirmed that apolipoprotein B (APOB) can induce calcification [7–10]. Lumican (LUM) is a functionally related extracellular matrix protein, which makes a profound impact upon the formation of the central spindle (with microtubules as the main element) and intermediates during cytokinesis [11]. LUM has been identified to be overexpressed in VMSCs with chronic renal failure [12]. LUM down-regulation also enhances mitotic defects and aneuploidy in lung cancer cells [13]. Therefore, we hypothesized that LUM/APOB/KIF2C axis may participate in the regulation of calcification in VMSCs.

In this study, we used β-glycerophosphate (β-GP) to trigger the calcification of human VMSCs (HVMSCs) and explored the role of LUM/APOB/KIF2C axis in the calcification of HVMSCs and the corresponding mechanism.

## 2 Materials and methods

### 2.1 Cell culture

HVSMCs (C1601) ordered from WHELAB (Shanghai, China) were incubated with complete Dulbecco’s modified eagle medium (DMEM) (M1023, WHELAB, China) in a cell incubator (3111, THERMO, MA, USA).

### 2.2 Transfection

The coding sequence of KIF2C (NM_006845) or APOB (NM_000384) was amplified by PCR and inserted into pcDNA3.1 vector (V87020, Invitrogen, USA) to construct pcDNA-KIF2C overexpression plasmid or pcDNA-APOB overexpression plasmid. The empty vector served as negative control (NC). Short hairpin RNA for LUM (shLUM, abx952749) and shNC (abx991273) were procured from Abbexa (USA). HVSMCs were transfected with pcDNA-KIF2C/pcDNA-APOB overexpression plasmid or shLUM or NC/shNC under the help of lipofectamine 3000 reagent (L3000-015, Invitrogen, USA) for 48 h. Quantitative real-time PCR (qRT-PCR) or Western blot experiments were further performed to measure transfection efficiency.

### 2.3 Cell treatment

HVSMCs were allocated into four groups: control group (cells without treatment); β-GP group (cells were treated with 10 mM β-GP (D301908, Aladdin, China) for 12 days [14]); and β-GP + NC/β-GP + KIF2C group (cells were transfected with NC or KIF2C overexpression plasmid prior to treatment with 10 mM β-GP for 12 days). HVSMCs were also distributed to the control group, the β-GP group, and the β-GP + shNC/β-GP + shLUM group (cells were transfected with shNC or shLUM before being treated with 10 mM β-GP for 12 days).

### 2.4 Alizarin red S staining

Alizarin red S staining, a commonly utilized method for calcium salt staining [15], was performed to determine the content of cellular calcium deposition. HVSMCs with different treatments were rinsed with phosphate-buffered saline and then fixed with 10% formalin (G2162; Solarbio, China) for 15 min. After washing, 0.2% alizarin red S solution (G1450; Solarbio, China) was applied to stain HVSMCs (0.5 h). The staining results were observed under an optical microscope (200×, DMi8; Leica Microsystems, Wetzlar, Germany).

### 2.5 Western blot

To obtain total protein in HVSMCs, cell lysate (PS0009; Leagene, China) was utilized. Next, bicinchoninic acid kit (PT0001; Leagene, China) was exploited to evaluate protein concentration. After denaturation and electrophoresis, the protein (30 μg) was blotted onto nitrocellulose membrane which was then sealed by 5% bovine serum albumin (R00911; Leagene, China) at 37℃ for 1 h. Later, the membrane was incubated with primary antibodies at 4℃ overnight and then with anti-rabbit secondary antibody at 37℃ for 1 h. The reactive bands were visualized with enhanced chemiluminescence kit (KGP1123, KeyGEN, China) under an Imaging System (Geliance 200; PerkinElmer, USA). GAPDH was validated as the normalizer. The information of antibodies is listed in Table 1.

**Table 1:** The information of antibodies

Antibodies	Cat no.	Dilution	Company
Anti-LUM	ab252925	1:1,000	Abcam
Anti-APOB	ab139401	1:50,000	Abcam
Anti-KIF2C	ab187652	1:2,000	Abcam
Anti-Osteocalcin	ab93876	1:1,000	Abcam
Anti-Runx2	ab264077	1:1,000	Abcam
Anti-α-SMA	ab5694	1:1,000	Abcam
Anti-GAPDH	ab181602	1:10,000	Abcam
Anti-rabbit secondary antibody	31466	1:8,000	Invitrogen

### 2.6 qRT-PCR

Total RNA was acquired from HVSMCs using RNA extraction kit (NE0260, Leagene, China). For qRT-PCR analysis, TaqMan One-Step RT-qPCR Kit (T2210, Solarbio, China) was employed. The reaction plate was amplified under a PCR machine (CFX96; Bio-Rad, USA). The qRT-PCR program was initiated with the reverse transcription reaction (50℃, 20 min) and denaturation (95℃, 3 min), followed by 35 cycles of denaturation at 95℃ for 20 s and annealing at 60℃ for 60 s. The reaction system was prepared based on the protocol of the One-Step RT-qPCR Kit. GAPDH was validated as the endogenous gene. Data were analyzed with the 2^−ΔΔCt^ method [16]. Table 2 presents the primers.

**Table 2:** Gene sequence primers

Name	Forward primer (5′–3′)	Reverse primer (5′–3′)
Lumican	GGATTGGTAAACCTGACCTTCAT	GATAAACGCAGATACTGCAATGC
APOB	TGAGGAGAAGAATCGAACCCT	CTTGATTTCGTAGAGCAGACAGG
KIF2C	CTCAGTTCGGAGGAAATCATGTC	TGCTCTTCGATAGGATCAGTCA
GAPDH	CTGGGCTACACTGAGCACC	AAGTGGTCGTTGAGGGCAATG

### 2.7 Quantification of calcium

Calcium assay kit (C004-2-1, Jiancheng, China) was used to examine the Ca^2+^ content in HVSMCs. HVSMCs with different intervention were collected and subsequently lysed and centrifuged (4℃, 12,000×*g*, 5 min). The supernatant was collected to measure Ca^2+^ content according to the instructions [17].

### 2.8 Assessment of alkaline phosphatase (ALP) activity

With the use of ALP assay kit (P0321M, Beyotime, China), ALP activity was determined. Briefly, HVSMCs were lysed according to the calcium content detection method, and then, the supernatant was harvested. Thereafter, ALP activity of HVSMCs was evaluated according to the instructions of ALP assay kit [18].

### 2.9 Immunofluorescence

HVSMCs (6000/well) were seeded into six-well plates with coverslip until reaching 80% confluence. After treatment, cells were fixed with 10% formalin, and permeabilized by 0.2% Triton X-100 (G5060; Servicebio, China). Next, cells were reacted with Alexa Fluor^®^ 647 Anti-Tubulin antibody (ab195884; Abcam, UK) at 4℃ overnight. A fluorescence microscope (200×, Ts2-FC; Nikon, Japan) was used to capture and observe the staining results.

For colocalization, cells were incubated with rabbit anti-LUM (ab168348, 1:250; Abcam) and mouse anti-APOB (GTX60445, 1:200; GeneTex) antibodies, followed by successive culture with secondary antibodies, Alexa Fluor^®^ 488 (ab150077; Abcam) and Alexa Fluor^®^ 647 (ab150115; Abcam). Nuclei were labeled with DAPI (ab228549; Abcam). Protein images were captured using Zeiss LSM 710 confocal microscope (Carl Zeiss AG, Oberkochen, Germany).

### 2.10 Examination of tubulin in HVSMCs

To analyze tubulin dynamics, the ratio of polymerized (Po) to free (Fr) tubulin was tested with Microtubule/Tubulin Biochem Kit (BK015, Cytoskeleton, USA). In short, HVSMCs were homogenized with tubulin buffer, centrifuged (100,000×*g*, 37℃, 0.5 h), and re-suspended with 200 μM CaCl_2_. Then, Po and Fr tubulin fractions were applied for Western blot assay.

### 2.11 Co-immunoprecipitation (Co-IP)

HVSMCs were lysed with cell lysis buffer and centrifuged to obtain the supernatant. The supernatant was successively reacted with anti-LUM antibody (1:30), anti-APOB antibody (MA5-14741; Invitrogen, USA), anti-IgG antibody (immunoglobulin G) (abx132131; Abbexa, UK), and Pierce™ Protein A/G Magnetic Beads (88802; Thermo Scientific, USA) at 4℃ overnight. Finally, the immunocomplex was collected and applied for Western blot assay.

### 2.12 Statistics

Data from triplicate independent experiments were described as mean ± standard deviation. GraphPad Prism 8.0 (PRISM 5.0, CA, USA) was employed for statistical analysis. Data comparisons between two groups were accomplished by independent samples *t*-test. Comparisons among multiple groups were completed by one-way analysis of variance with Tukey’s post hoc test. Statistical significance was accepted when *P* < 0.05.

## 3 Results

### 3.1 β-GP promoted HVSMC calcification and the expressions of LUM and APOB while repressing KIF2C expression

**Figure 1:**
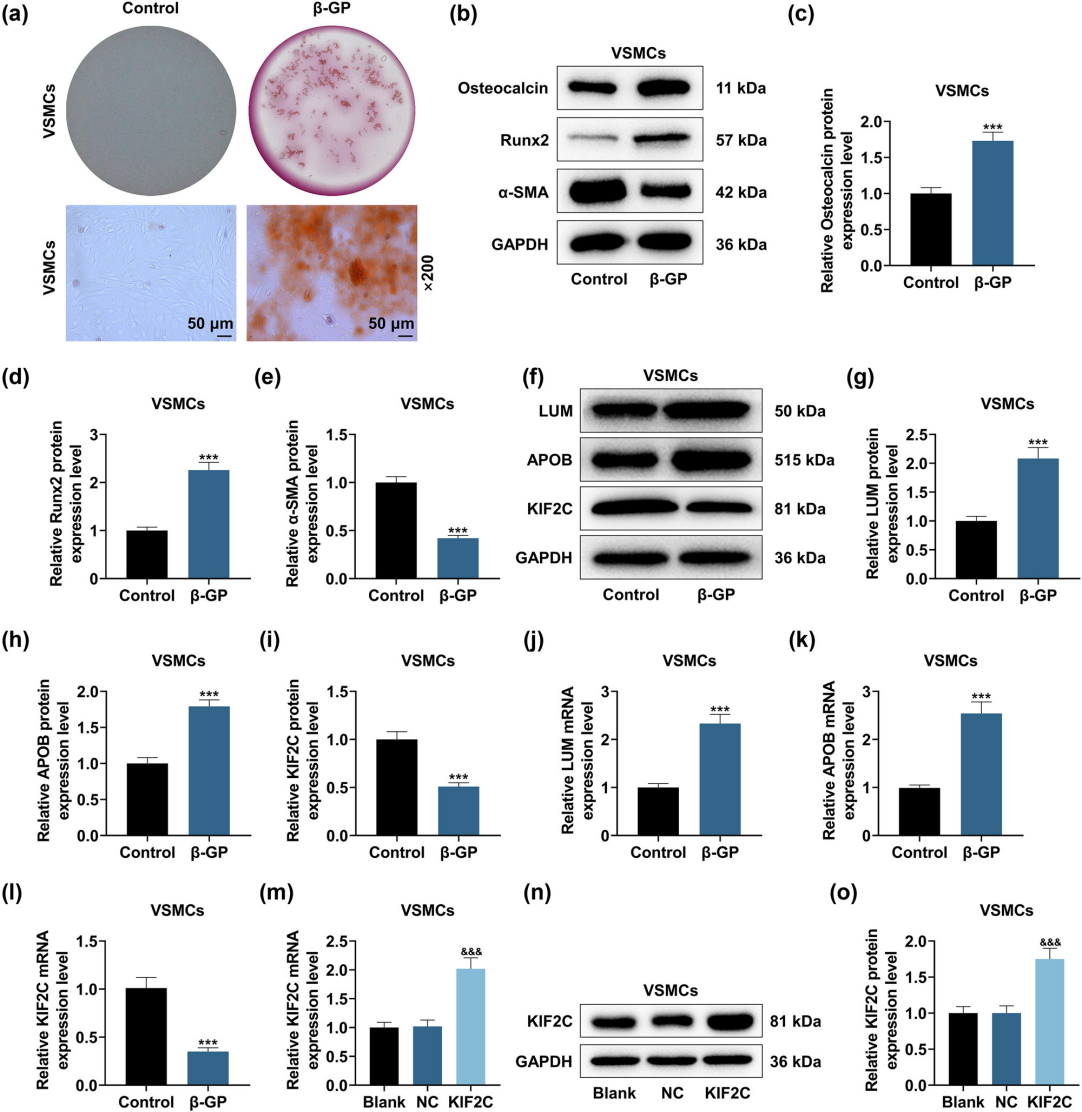
β-GP promoted HVSMC calcification and the expressions of LUM and APOB while repressing KIF2C expression. (a) Vascular calcification (VC) was measured with the help of Alizarin red S staining (200×, 50 μm). (b–e) The calcification-related markers were quantified by Western blot. GAPDH was validated as the endogenous gene. (f–l) The levels of lumican (LUM), apolipoprotein B (APOB), and kinesin family member 2C (KIF2C) were examined by Western blot and qRT-PCR. GAPDH was validated as the endogenous gene. (m–o) The level of KIF2C in human vascular smooth muscle cells (HVSMCs) transfected with KIF2C overexpression plasmid or its negative control (NC) was examined via qRT-PCR and Western blot, with GAPDH as the normalizer. ***P < 0.001 vs control; ^&&&^ *P* < 0.001 vs NC. *N* = 3. Data comparisons between two groups were completed by independent samples *t*-test. β-glycerophosphate (β-GP) concentration: 10 mM.

Alizarin red S staining results in Figure 1a displayed that β-GP led to HVSMC calcification. Next, levels of calcification-associated markers were measured. The up-regulation of Osteocalcin and Runx2 and the down-regulation of α-SMA were found in HVSMCs mediated by β-GP (Figure 1b–e, *P* < 0.001). Furthermore, elevated mRNA and protein levels of LUM and APOB and reduced KIF2C level were observed in HVSMCs mediated by β-GP (Figure 1f–l, *P* < 0.001). In addition, the increased KIF2C level was indicative of success transfection of KIF2C overexpression plasmid into HVSMCs (Figure 1m–o, *P* < 0.001).

### 3.2 The modulation of β-GP on HVSMC calcification, Ca^2+^ content, ALP activity, and microtubule cytoskeleton was reversed by KIF2C overexpression

**Figure 2:**
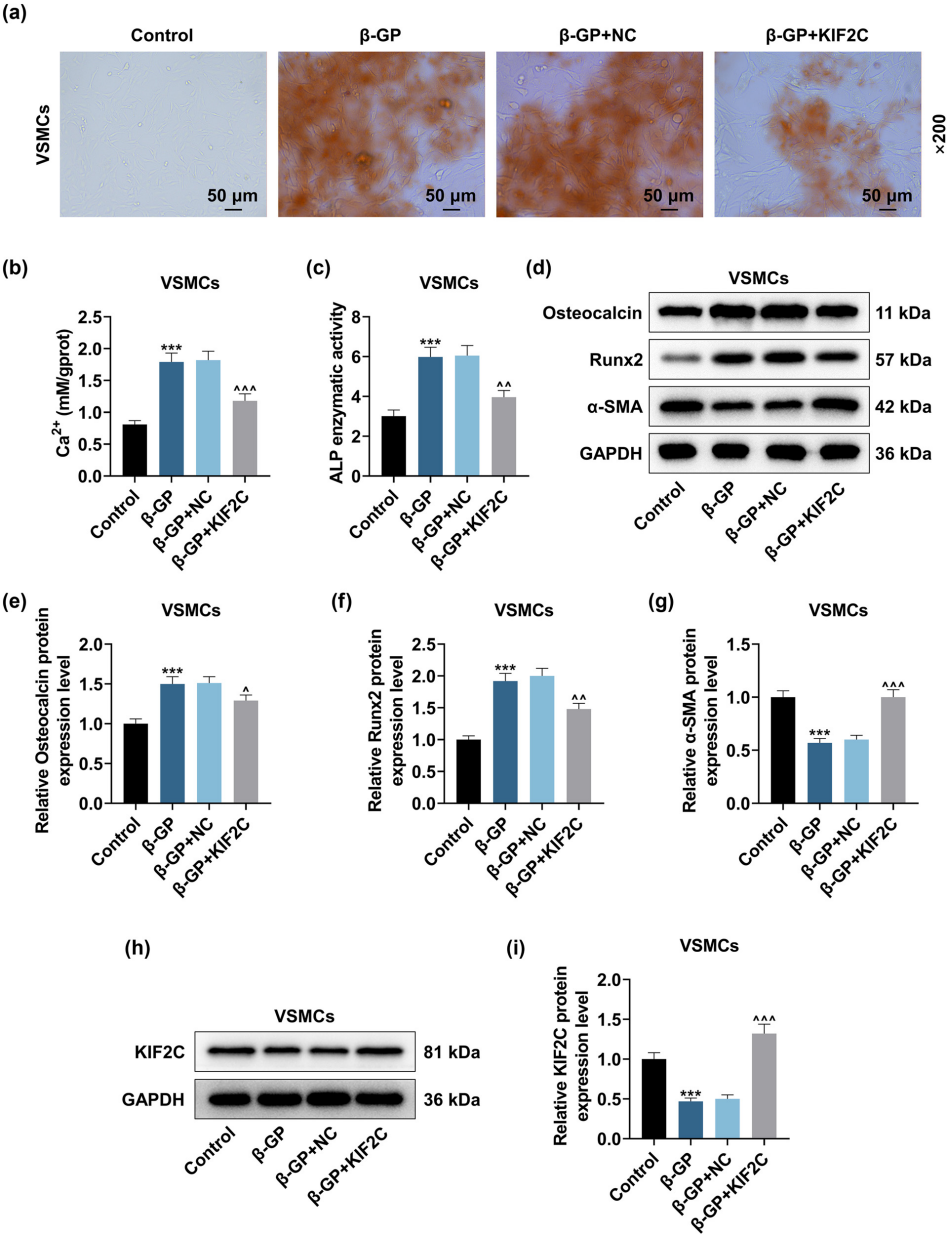
The modulation of β-GP on HVSMC calcification, Ca^2+^ content, ALP activity, and calcification-related marker was reversed by KIF2C overexpression. The impacts of KIF2C overexpression upon β-GP-induced HVSMC calcification (a), Ca^2+^ content (b), alkaline phosphatase (ALP) activity (c), and KIF2C and calcification-related marker expressions (d–i) were examined by Alizarin red S staining, calcium assay kit, ALP assay kit and Western blot. GAPDH was validated as the endogenous gene. ***P < 0.001 vs control; ^^^ *P* < 0.05, ^^^^ *P* < 0.01, ^^^^^ *P* < 0.001 vs β-GP + NC. *N* = 3. Comparisons among multiple groups were completed by one-way analysis of variance with Tukey’s post hoc test.

**Figure 3:**
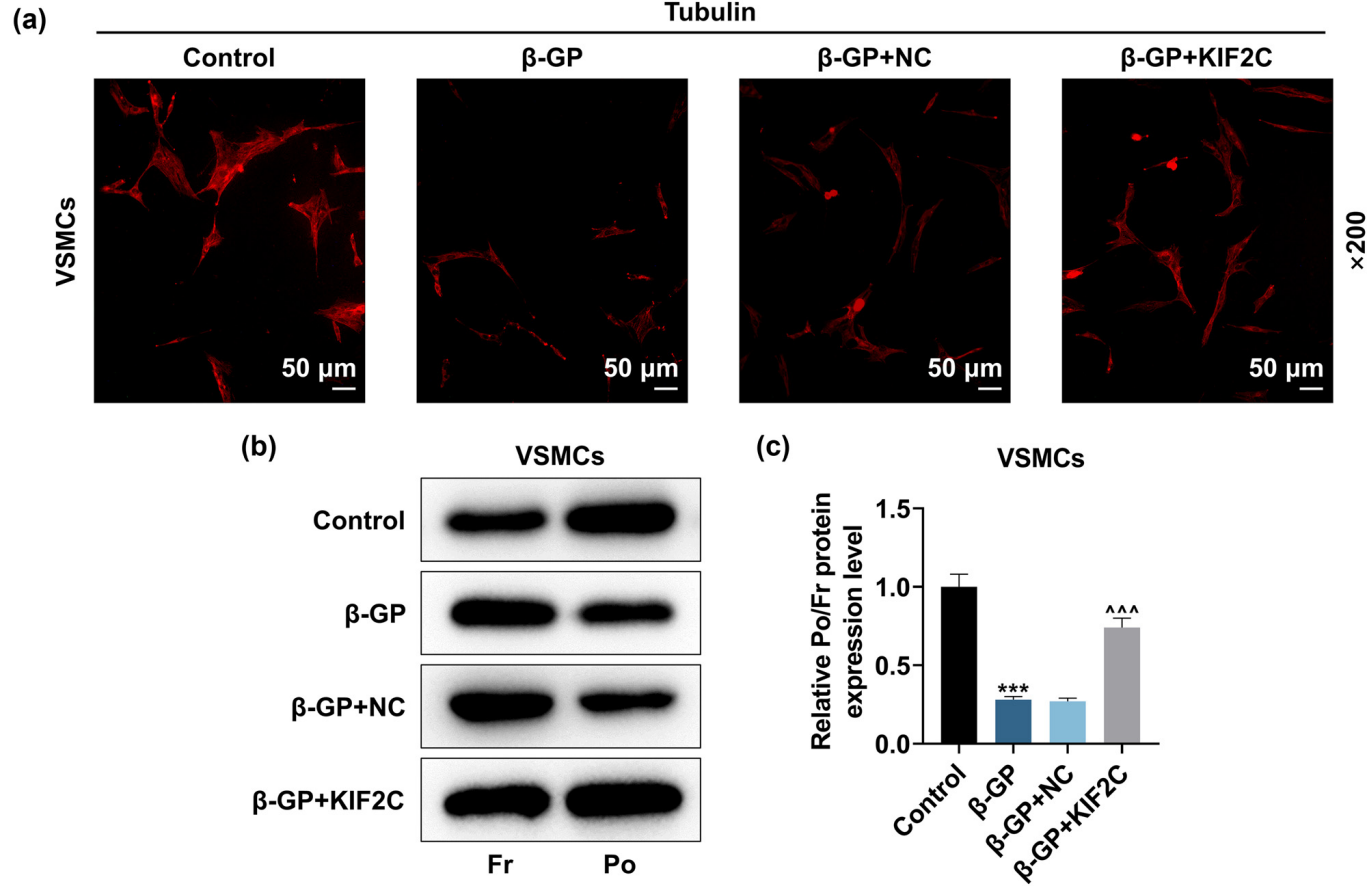
The impacts of KIF2C overexpression upon microtubule cytoskeletons in HVSMCs. Tubulin expression (a) and the ratio of polymerized (Po) to free (Fr) tubulin (b and c) were examined by immunofluorescence and Western blot. ***P < 0.001 vs control; ^^^^^ *P* < 0.001 vs β-GP + NC. *N* = 3. Comparisons among multiple groups were completed by one-way analysis of variance with Tukey’s post hoc test.

After transfection, we discovered that KIF2C up-regulation decreased β-GP-induced HVSMC calcification (Figure 2a). Next, we examined Ca^2+^ content and ALP activity utilizing the corresponding kits. KIF2C up-regulation has been identified to effectively reduce Ca^2+^ content and ALP activity in HVSMCs induced by β-GP (Figure 2b and c, *P* < 0.01). KIF2C overexpression reversed the inhibitory effect of β-GP on KIF2C and α-SMA protein levels and also strongly decreased Osteocalcin and Runx2 levels in β-GP-induced HVSMCs (Figure 2d–i, *P* < 0.05). To reveal the possible relation between VC and microtubule cytoskeletal reorganization, we determined the dynamics of tubulin in VSMCs exposed to β-GP using fluorescence microscopic analysis and measured the ratio of Po/Fr tubulin. The immunofluorescence staining results indicated that β-GP inhibited the positive expression of Tubulin, which was reversed by KIF2C up-regulation (Figure 3a). β-GP reduced the ratio of Po/Fr tubulin in HVSMCs, the effect of which was partially reversed by KIF2C overexpression (Figure 3b and c, *P* < 0.001). These findings indicated that microtubules were disrupted during calcification of HVSMCs, which was reversed by KIF2C overexpression.

### 3.3 LUM silencing neutralized the regulation of β-GP on HVSMC calcification, Ca^2+^ content, ALP activity, APOB expression, and KIF2C expression

**Figure 4:**
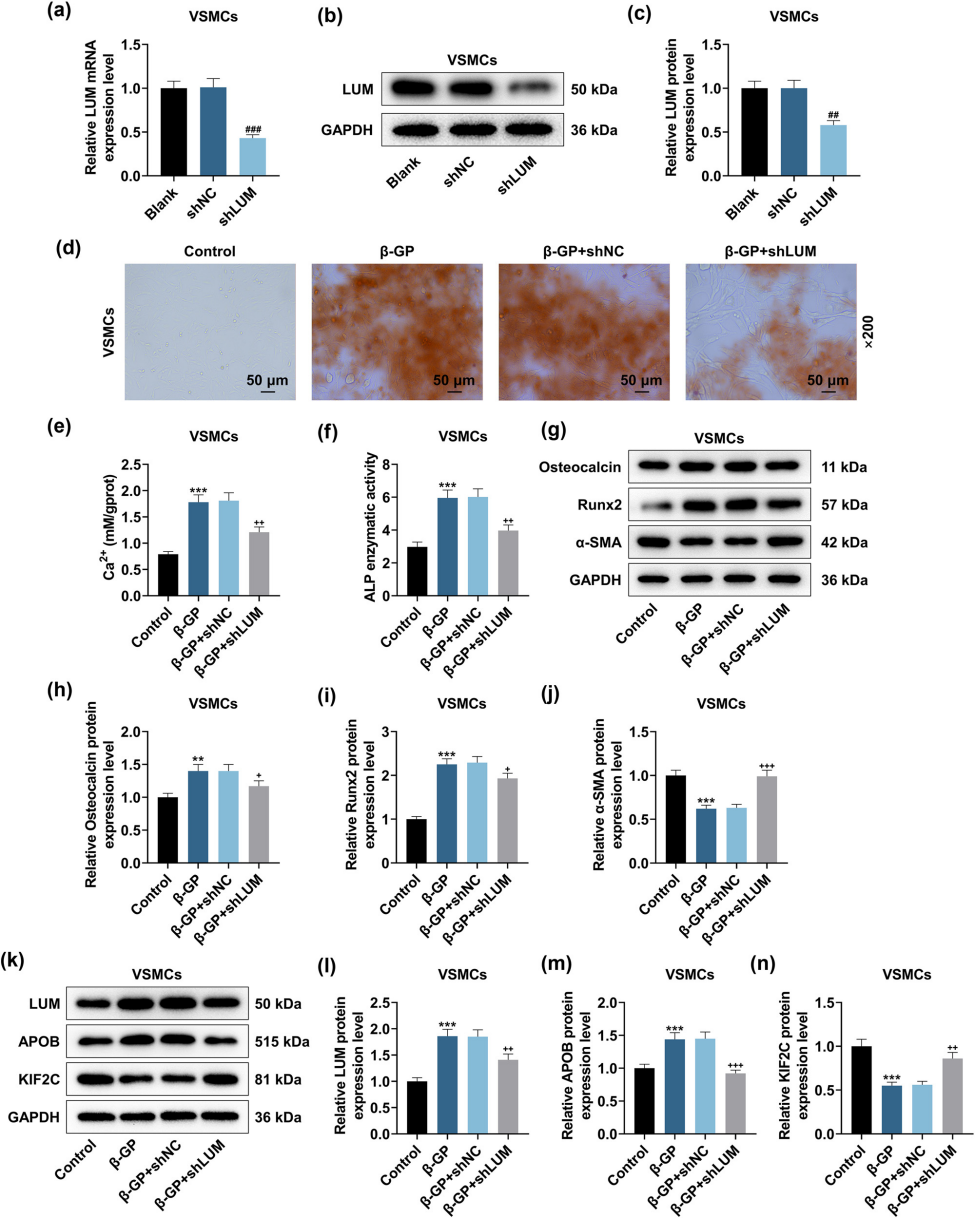
LUM silencing attenuated the regulation of β-GP on HVSMC calcification, Ca^2+^ content, ALP activity, APOB expression, and KIF2C expression. (a–c) The level of LUM in HVSMCs transfected with short hairpin RNA for LUM (shLUM) or its NC (shNC) was assessed by qRT-PCR and Western blot, with GAPDH as the normalizer. The impacts of shLUM upon β-GP-mediated HVSMC calcification (d), Ca^2+^ content (e), ALP activity (f), calcification-related marker expressions (g–j), LUM and APOB and KIF2C expressions (k–n) were determined by Alizarin red S staining, calcium assay kit, ALP assay kit, and Western blot. GAPDH was validated as the normalizer. ^##^ *P* < 0.01, ^###^ *P* < 0.001 vs shNC; **P < 0.01, ***P < 0.001 vs control; ^+^ *P* < 0.05, ^++^ *P* < 0.01, ^+++^ *P* < 0.001 vs β-GP + shNC. *N* = 3. Comparisons among multiple groups were completed by one-way analysis of variance with Tukey’s post hoc test.

Transfection of shLUM dwindled LUM expression in HVSMCs, proving that the transfection was successful (Figure 4a–c, *P* < 0.001). LUM silencing also counteracted the promoting effects of β-GP on HVSMC calcification, Ca^2+^ content, and ALP activity (Figure 4d–f, *P* < 0.01). Further, Western blot experiment results validated that LUM silencing remarkably suppressed the protein expressions of LUM, Osteocalcin, Runx2, and APOB and potentiated α-SMA and KIF2C protein expressions in β-GP-mediated HVSMCs (Figure 4g–n, *P* < 0.05).

### 3.4 LUM silencing increased KIF2C expression by interacting with APOB

**Figure 5:**
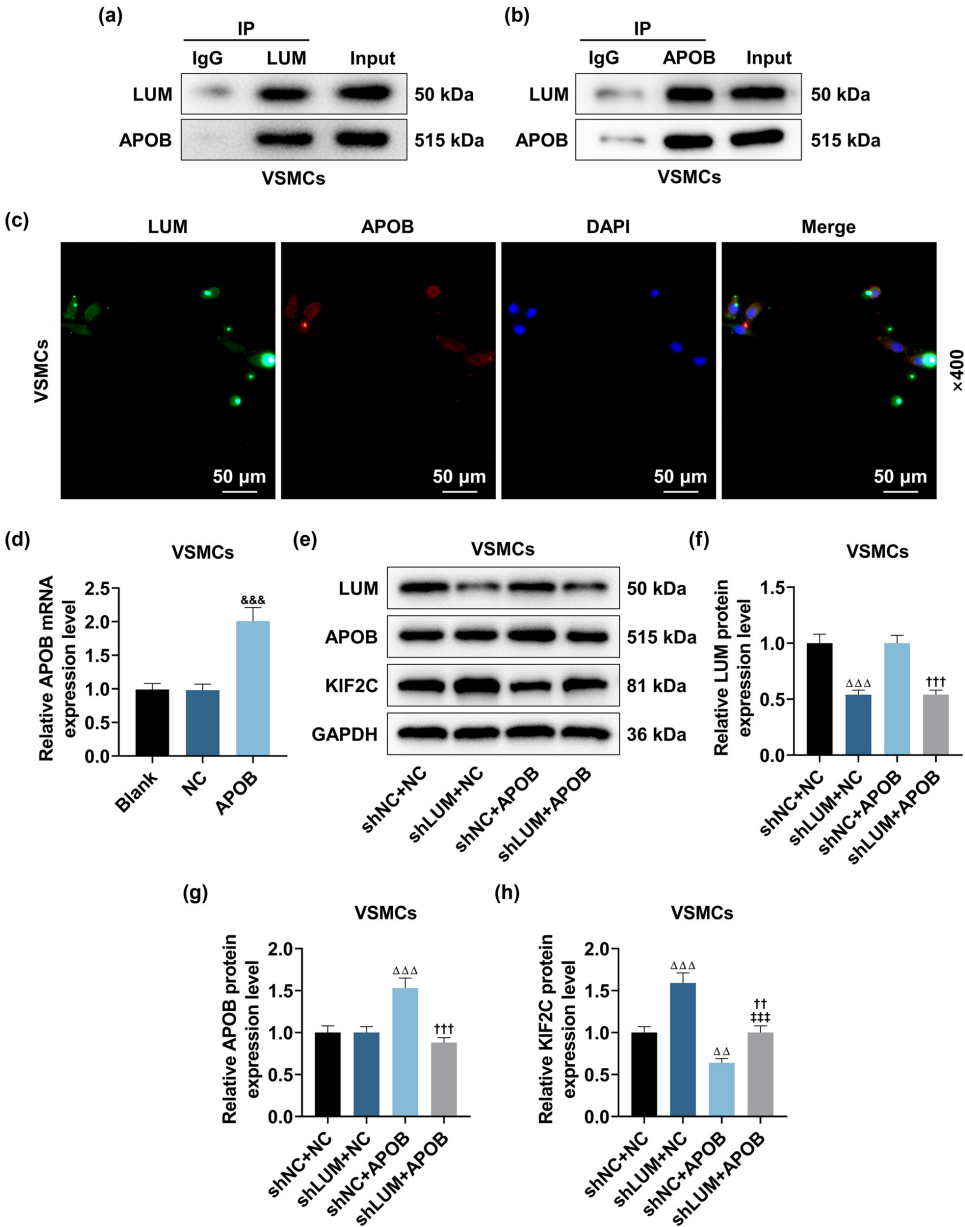
LUM silencing increased KIF2C expression by interacting with APOB. (a and b) The interplay between LUM and APOB was estimated through Co-immunoprecipitation (Co-IP). (c) The images of LUM (green) and APOB (red) expressions in HVSMCs revealed that LUM and APOB colocalized with each other. (d) The level of APOB in HVSMCs transfected with APOB overexpression plasmid was assessed by qRT-PCR, with GAPDH as the normalizer. (e–h) The effects of LUM and APOB on expressions of LUM, APOB, and KIF2C in HVSMCs were estimated using Western blot. GAPDH was validated as the housekeeping gene. ^&&&^ *P* < 0.001 vs NC; ^ΔΔ^ *P* < 0.01, ^ΔΔΔ^ *P* < 0.001 vs shNC + NC; ^††^ *P* < 0.01, ^†††^ *P* < 0.001 vs shNC + APOB; ^‡‡‡^ *P* < 0.001 vs shLUM + NC. *N* = 3. Comparisons among multiple groups were completed by one-way analysis of variance with Tukey’s post hoc test.

Next, we detected the interplay between LUM and APOB using Co-IP and immunofluorescence staining. As presented in Figure 5a–c, LUM and APOB formed a complex and interacted with each other in HVSMCs. Besides, HVSMCs transfected with APOB overexpression plasmid presented the high expression of APOB (Figure 5d, *P* < 0.001). According to Figure 5e–h, LUM silencing promoted while APOB overexpression inhibited KIF2C expression in HVSMCs (*P* < 0.001). More importantly, APOB overexpression partially offset the promoting effect of LUM silencing on KIF2C expression in HVSMCs (Figure 5e–h, *P* < 0.001) but did not affect the inhibiting effect of LUM silencing on LUM expression (Figure 5e–h, *P* < 0.001).

## 4 Discussion

Our findings provided new mechanistic information on the crucial role of KIF2C in VC process. We revealed that LUM was highly expressed in calcified HVSMCs, accompanied by increased level of APOB and decreased level of KIF2C. Besides, we found that LUM silencing ameliorated β-GP-induced HVSMC calcification and osteogenic differentiation through increasing KIF2C expression by interacting with APOB.

Both preclinical and clinical studies confirmed that dietary phosphorus restriction and the use of phosphorus binders can prevent hyperphosphatemia and weaken VC development [19,20]. The molecular mechanisms and therapeutic targets of VC are worthy of exploration. The transdifferentiation of VSMCs into osteoblast-like cells involves the production of a mass of calcium salts [21]. We discovered that more severe calcified nodules appeared in HVSMCs induced by β-GP, indicating that HVSMC calcification was successfully induced. At present, it is considered that the phenotypic transformation of VSMCs in the middle layer of blood vessels to osteoblasts is a primary factor leading to VC [22]. This phenotypic transdifferentiation is characterized by the loss of contraction-related proteins and the accumulation of osteoblast-related proteins, including Runx2, ALP, and osteocalcin [23]. Runx2 is a marker of phenotypic transformation in VSMCs, and ALP is a significant biomarker of calcification in VSMCs [24]. Osteocalcin, as a marker for evaluating bone formation, plays a primary role in the process of VC [25]. It has been documented that osteocalcin overexpression elevates the expression of Runx2, thereby inducing calcification in VSMCs [26]. Also, Runx2 expression is increased in VSMC calcification model induced by inorganic phosphate and calcium chloride [27]. In addition, when the expression of Runx2 was suppressed, VC was attenuated, together with down-regulated Osteocalcin [28]. In our study, we detected up-regulation of ALP, Osteocalcin and Runx2, and down-regulation of α-SMA in HVSMCs mediated by β-GP, coinciding with results of former reports [14,29].

KIF2C has been demonstrated to play a crucial role in various diseases, such as hepatocellular carcinoma, endometrial carcinoma, breast cancer, and bladder cancer [30,31]. KIF2C also can modulate microtubule dynamics [32,33]. Intriguingly, microtubule stabilization attenuates VC by inhibiting osteogenic signaling and matrix vesicle release [5]. Our study was the first to elucidate the effect of KIF2C on β-GP-triggered calcification in HVSMCs. The results proved that KIF2C overexpression reversed the modulation of β-GP on HVSMC calcification, Ca^2+^ content, ALP activity, calcification-related markers, and microtubule cytoskeleton (the ratio of Po to Fr tubulin). According to a previous report, stabilization of a curved tubulin contributes to the KIF2C mechanism [34]. These findings suggested that KIF2C up-regulation repressed the calcification of HVSMCs through mediating microtubule cytoskeleton and osteogenic induction. Currently, no study has reported the interplay between LUM and VC. The ectopic expression of LUM has been widely demonstrated to be associated with pathological processes, such as cardiac fibrosis, cardiomyocyte hypertrophy, and heart failure as well as in VMSCs with chronic renal failure [12,35–37]. Besides, LUM is activated as a tubulin-binding protein through interacting with microtubule-modulated p120ctn signaling [38]. Our study reported that LUM silencing reversed the regulation of β-GP on HVSMC calcification, Ca^2+^ content, ALP activity, calcification-related markers, APOB expression, and KIF2C expression. In a previous study, LUM promotes preosteoblast differentiation, which leads to increased calvaria bone formation, accompanied by up-regulation of osteoblast differentiation markers [39]. In our study, the findings indicated that LUM silencing can protect against VC by inhibiting transdifferentiation of VSMCs from contractile to osteogenic phenotype. APOB can be used to diagnose coronary artery calcification [40], and our study confirmed the effect of APOB in the calcification of HVSMCs. To further verify the interplay between LUM and APOB, we conducted Co-IP and immunofluorescence cell staining assays. The results revealed that LUM and APOB formed a complex and interacted with each other in HVSMCs. More importantly, APOB overexpression partially offset the promoting effect of LUM silencing on KIF2C expression in HVSMCs. In the future, an *in vivo* VC model should be established to verify the accuracy of the results in this study.

## 5 Conclusion

This study demonstrates that LUM silencing alleviates the calcification of β-GP-induced HVSMCs through attenuating the repression of APOB on KIF2C expression, thus providing a theoretical basis for the clinical treatment of VC associated with cardiovascular adverse events.
